# Hybrid Additive Fabrication of a Transparent Liver with Biosimilar Haptic Response for Preoperative Planning

**DOI:** 10.3390/diagnostics11091734

**Published:** 2021-09-21

**Authors:** Paolo Aseni, Tommaso Santaniello, Francesco Rizzetto, Lorenzo Gentili, Federico Pezzotta, Francesco Cavaliere, Maurizio Vertemati, Paolo Milani

**Affiliations:** 1 Department of Emergency, ASST Grande Ospedale Metropolitano Niguarda, Piazza Ospedale Maggiore 3, 20162 Milano, Italy; paoloaseni@gmail.com; 2 Department of Biomedical and Clinical Sciences “L. Sacco”, Università degli Studi di Milano, Via Giovanni Battista Grassi 74, 20157 Milano, Italy; 3 Centro Interdisciplinare Materiali e Interfacce Nanostrutturati (CIMaINa), Università degli Studi di Milano, Via Celoria 16, 20133 Milano, Italy; tommaso.santaniello@unimi.it (T.S.); lorenzo.gentili@unimi.it (L.G.); federico.pezzotta@unimi.it (F.P.); francesco.cavaliere@unimi.it (F.C.); 4 Dipartimento di Fisica “A. Pontremoli”, Università degli Studi di Milano, Via Celoria 16, 20133 Milano, Italy; 5 Department of Radiology, ASST Grande Ospedale Metropolitano Niguarda, Piazza Ospedale Maggiore 3, 20162 Milano, Italy; francesco.rizzetto@unimi.it; 6 Postgraduate School of Diagnostic and Interventional Radiology, Università degli Studi di Milano, Via Festa del Perdono 7, 20122 Milano, Italy

**Keywords:** additive manufacturing, 3D organ models, haptic feedback, anatomical liver resection, surgical training, preoperative planning

## Abstract

Due to the complexity of liver surgery, the interest in 3D printing is constantly increasing among hepatobiliary surgeons. The aim of this study was to produce a patient-specific transparent life-sized liver model with tissue-like haptic properties by combining additive manufacturing and 3D moulding. A multistep pipeline was adopted to obtain accurate 3D printable models. Semiautomatic segmentation and registration of routine medical imaging using 3D Slicer software allowed to obtain digital objects representing the structures of interest (liver parenchyma, vasculo-biliary branching, and intrahepatic lesion). The virtual models were used as the source data for a hybrid fabrication process based on additive manufacturing using soft resins and casting of tissue-mimicking silicone-based blend into 3D moulds. The model of the haptic liver reproduced with high fidelity the vasculo-biliary branching and the relationship with the intrahepatic lesion embedded into the transparent parenchyma. It offered high-quality haptic perception and a remarkable degree of surgical and anatomical information. Our 3D transparent model with haptic properties can help surgeons understand the spatial changes of intrahepatic structures during surgical manoeuvres, optimising preoperative surgical planning.

## 1. Introduction

Over the last decade, increasing attention has been focused on three-dimensional (3D) printing of organ models for surgical simulation and training [1,2,3,4]. Musculoskeletal [5,6,7,8], neurological [9,10], cranio-maxillo-facial [11,12], genitourinary [13,14], and cardiovascular [15,16] surgery are just some of the areas where the clinical application of 3D printing is constantly increasing. As proof of the impact expected on patient care and management, in 2019, the Radiology Society of North America (RSNA) and the American College of Radiology launched a joint data registry to collect 3D printing information and answer questions about technical specifications and clinical indications for this new technology [17]. Recommendations and guidelines were also published to coordinate the standardisation of 3D printing in healthcare and optimise resource utilisation [18,19].

This mounting interest in 3D printing stems from the possibility to use a computed tomography (CT) and magnetic resonance imaging (MRI) to obtain 3D models of almost any organ with a high degree of accuracy. The improved spatial and contrast resolution of radiological images and the rapid advances in additive manufacturing technologies allow printing anatomical and pathological structures with accurate patient-specific details. These advantages make 3D printing a valuable tool for the preoperative planning of surgical procedures and training purposes [20].

A field that can particularly benefit from the use of 3D printed phantoms is hepatobiliary (HB) surgery. The surgical treatment of hepatocellular carcinoma, colorectal liver metastasis, traumatic lesions, and complex congenital biliary cysts are some scenarios where preoperative planning is crucial to minimise the removal of healthy liver parenchyma, reducing the risk of postoperative liver failure [21].

HB surgeons must choose the operative strategy along different anatomical planes depending on the relationship of the liver lesion with the different and variable patterns of the vasculo-biliary branching. This task can be challenging due to the high complexity of liver anatomy, and severe life-threatening complications may occur, such as extensive bleeding and large areas of hepatic ischemia or infarction [22,23,24]. For this reason, the RSNA guidelines indicate as appropriate the use of 3D printed models for the surgical management of hepatic masses, as it extends the value of the information contained in medical images [18]. Similarly, 3D printed liver models have been used for preoperative planning and risk assessment of liver transplant candidates [25]. Given the peculiar characteristics of the hepatic parenchyma and its internal structures, the implementation of 3D printable materials capable of delivering a life-like tactile response would further boost the value of this technology in HB surgery [26,27,28,29,30].

Additive manufacturing technologies relying on the use of material jetting are reported in the literature as the most suitable solution for the direct 3D printing of monolithic complex structures with multi-coloured features. They have already been used in several case studies related to hepatic surgery [25,31]. However, implementing these solutions in daily clinical practice is still burdensome due to the use of expensive printers, long processing time, and sophisticated software that are difficult to be available in hospitals. Moreover, the 3D-printed anatomical models produced with these approaches do not reproduce the deformability properties and haptic feedback of human tissues, hindering the simulated experience of surgical manipulation.

In this work, a multistep pipeline from the semiautomatic annotation of standard CT and MRI scans to the hybrid additive fabrication of a 3D patient-specific transparent liver model with an intra-parenchymal lesion with haptic properties is presented. The liver physical model was realised by combining 3D printing technologies based on soft photopolymers to produce the vascular and biliary branching and 3D moulding to reproduce the parenchyma, employing a tissue-equivalent silicone developed in-house. We targeted at the production of a system enabling better diagnostic preoperative planning before or during hepatobiliary surgical procedures due to its transparency and superior haptic properties. The aim of this paper was to investigate how the 3D liver model could be used as an effective simulation tool for medical and surgical training, with potential to help surgeons to understand the spatial changes of intrahepatic structures during surgical manoeuvres, with a possibility to improve the safety of complex liver surgery.

The simulated liver properties were evaluated by a board of clinicians (radiologists and HB surgeons); they were asked to give their evaluation by a scoring system based on the assessment of haptic properties of the liver phantom and its capability to improve the comprehension of the anatomical details and spatial relations of the vasculo-biliary structures when compared with the standard imaging techniques.

## 2. Materials and Methods

### 2.1. Image Processing and 3D Reconstruction of the Target Anatomy

The fabricated haptic model was based on a virtual model obtained from routine CT and MRI images in which the anatomical structures were appropriately segmented, i.e., labelled to distinguish them from the background and other structures of interest. For both imaging techniques, the field of view of the image acquisition included the liver and its intra-parenchymal structures, its bilio-vascular pedicle (aorta with celiac trunk and hepatic artery, main portal vein, and the extrahepatic biliary system), and its venous drainage (hepatic veins and inferior cava vein).

The segmentation of the different anatomical structures was obtained by matching the specific morphological information given by the different modalities, contrast phases, or imaging sequences. In detail, iodine contrast-enhanced 64-slice CT scans were acquired in the arterial and venous phases and were reconstructed with a slice thickness of 1 mm on the axial, coronal, and sagittal plane. Hepatic arteries were segmented using the arterial phase, while liver parenchyma, portal branching, and hepatic veins were segmented using the portal venous phase.

MRI scans were performed in a 1.5 T scanner, and axial and coronal 3D SENSE sequences acquired 90 min after administration of hepatobiliary contrast agent with a slice thickness of 1.5 mm were used for the segmentation of the biliary system. Images from multislice 2D balanced turbo-field-echo sequence with a slice thickness of 2.5 mm were also considered for integration in case of uncertain findings.

The aforesaid CT and MRI images were retrieved from the institutional PACS (Picture Archiving and Communication System), anonymised, and uploaded into 3D Slicer v.4.11), a free and open-source software package for image analysis and scientific visualisation. Image rigid co-registration and volumetric segmentation were performed with pre-built functions in the software. When necessary, the automatic and semiautomatic segmentations were manually corrected under the supervision of an experienced radiologist.

**Figure 1:**
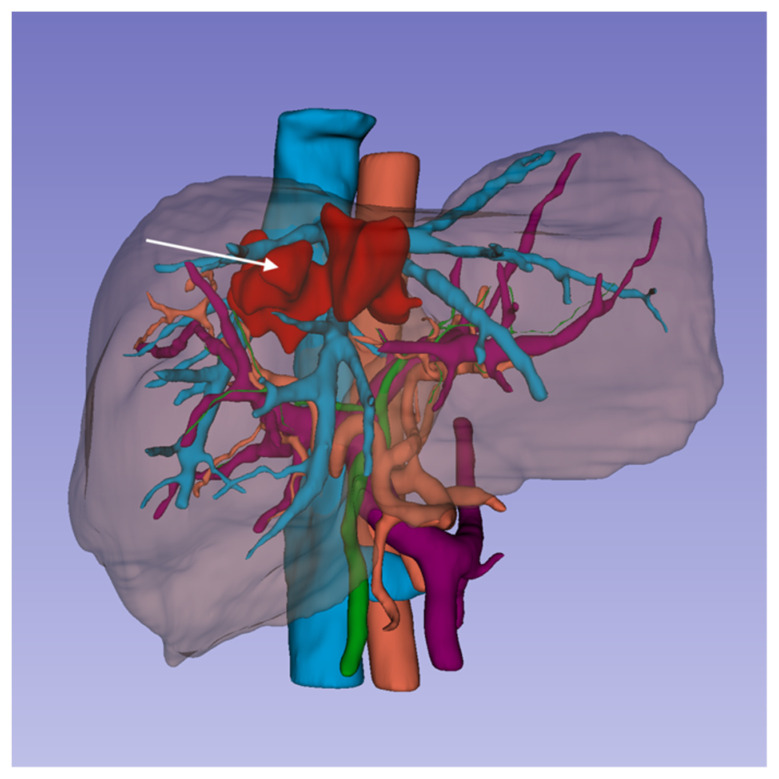
3D surface models of liver parenchyma and its bilio-vascular structures with the intrahepatic lesion (right lobe hematoma, white arrow) obtained from segmentation of medical imaging. The virtual models were exported in STL files for 3D printing.

The finally approved segmentations (Figure 1) were exported into StereoLithography (.stl) files for the following steps of the fabrication pipeline.

### 2.2. 3D Haptic Liver Fabrication: Rationale and General Approach

**Figure 2:**
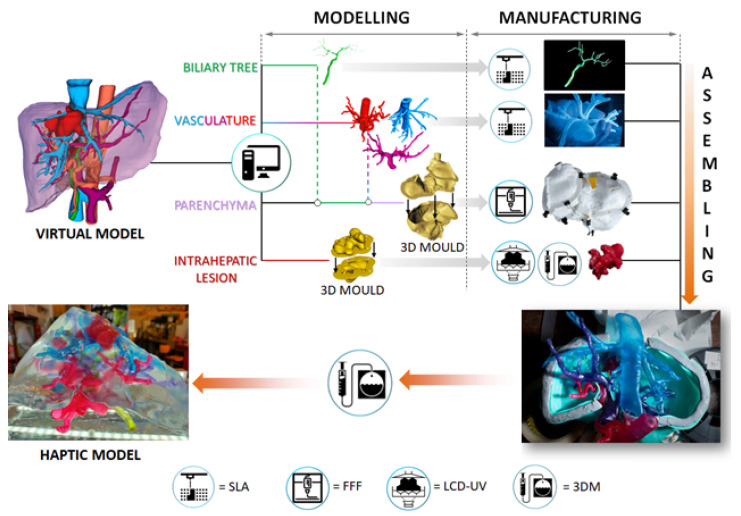
Schematic of the workflow adopted for the fabrication of the haptic liver models. Legend: SLA = stereolithography; FFF = fused filament fabrication; LCD-UV = liquid crystal display based VAT photopolymerisation; 3DM = 3D moulding.

We used a hybrid fabrication approach to produce the haptic liver models based on additive manufacturing and casting of tissue-mimicking materials into three-dimensional moulds. The core of this methodology is based on the 3D assembly of the physical models of the vasculo-biliary branching and intrahepatic lesion into a dedicated mould reproducing the morphology of the parenchyma. After assembling the system keeping the correct anatomical configuration and relative spatial features (i.e., distances between vascular branches, mutual position between vascular and biliary trees), a tissue-equivalent silicone-based blend, developed in-house, was poured into the mould, forming the parenchymal structure embedding the arterio-venous intrahepatic branching, biliary duct, and lesion. The result is a life-sized, anatomically realistic, and haptic transparent 3D liver model (total organ weight equal to 1.47 kg, with parenchyma mass density equal to 0.98 g/cm^3^). The general workflow that we adopted to obtain the fully assembled prototype starting from the digital models is reported in Figure 2.

We fabricated hollow vascular structures in view of contrast media injection for metrological validation of the prototypes to provide a realistic version of the organ anatomy [32]. The native models of the vessels were processed using the free software Autodesk Meshmixer (http://www.meshmixer.com, accessed on 18 September 2021) to generate a 1 mm thick wall around the venous and arterial lumen, which was then eliminated using Boolean subtractions to obtain the empty vessels. The biliary tree was only slightly refined to eliminate the terminal ducts with a diameter below 0.2 mm, maintaining the solid structure and branching geometry.

The refined models were then used in conjunction with that of the parenchyma to design and generate a 3D mould for silicone casting. This was engineered as a four part system that can be joined together using mechanical fastening and provided with alignment and positioning channels able to host the vascular and biliary ducts, keeping their original spatial configuration and relative distances between branches. The mould was also endowed with flanges to favour the four components assembly with a similar approach; the intrahepatic lesion model was used to generate a 3D mould for silicone casting of the pathology, consisting of a two components system that can be interlocked together.

### 2.3. Phantom Fabrication

#### 2.3.1. Vessels and Biliary Duct 3D Printing

The vascular structures and biliary tree were directly printed by means of stereolithographic 3D printing using low hardness commercial photopolymers, employing a Form2 printing apparatus (Formlabs), equipped with a 405 nm wavelength laser [32]. The orientation of the different parts with respect to the printing plate was carefully selected using the printer slicer to optimise the number and configuration of supports. The hollow portal and hepatic veins as well as hepatic arteries, were fabricated using a 50 ShoreA and 3.23 MPa ultimate tensile strength resin (Elastic, Formlabs) to reproduce the haptic response of the biological vessels walls [33,34], while the solid biliary tree, characterised by small-diameter branches (0.3 to 1.5 mm), was produced using an 80 ShoreA photopolymer (Flexible, Formlabs). Layer height was set to 0.15 mm for all the manufacturing processes to achieve a smooth surface finish of the printed parts and relatively short fabrication times (between 6 and 10 h, depending on the specific piece). After printing, the objects were thoroughly washed and rinsed in 2-propanol for 30 min. For the printed vessels, the washing operation was conducted with the help of a syringe to remove the unreacted resin from the ducts. The post-curing procedure took place at 50 °C for 1 h under UV light exposure to promote the complete polymerisation of the pieces. Supports were then manually removed using trimming scissors. The different structures were coloured according to the colour code of the digital model’s representation reported in Figure 2 (red, light blue, purple and green for the hepatic arteries, veins, portal veins, and biliary tree, respectively) by soaking them into alcoholic solutions containing aniline-based pigments.

#### 2.3.2. Intrahepatic Lesion Moulding

The intrahepatic lesion mould was manufactured using LCD-UV 3D printing, employing an 82 ShoreD hard photopolymer (Light Grey Resin from 3Djake) with high dimensional stability and low shrinkage after curing. The printer used was a Kentstrapper Aura (Kentstrapper srl, Florence, Italy), equipped with a 4K LCD projector and 405 nm UV photodiodes. We used the Key-to-Box slicer to set the printing parameters (layer height equal to 0.15 mm and layer exposure time equal to 10 s). The washing, post-curing and support removal procedures were carried out as previously described. We moulded the malformation using a 20 Shore00 platinum cured silicone (Ecoflex, Smooth-On), coloured with a mix of red and brown pigments. The result is a soft 3D structure morphologically identical to the lesion that was then manually positioned with respect to the intrahepatic veins model using 0.2 mm thin nylon threads.

#### 2.3.3. 3D Assembling and Parenchyma Moulding

The 3D mould for the structures assembly and parenchyma fabrication was performed with a fused filament fabrication (FFF), using acrylonitrile butadiene styrene (ABS). Two printers were used, in parallel, to minimise fabrication times: a Delta 2040 (Wasp) and a 3DL Cartesian machine (Zeus). The nozzle diameter was 0.4 mm in both cases. We employed Simplify3D as the slicing software and set the following fabrication parameters: (i) extrusion temperature = 240 °C; (ii) printing bed temperature = 110 °C; (iii) layer height = 0.2 mm; (iv) printing speed equal to 40 mm/s. After support removal, we performed an acetone smoothening of the component’s inner walls using a set of soft brushes to minimise the high surface roughness typical of the FFF manufactured pieces, so to favour the parenchyma transparency after moulding.

After assembling the different anatomical parts into the 3D mould, it was encapsulated using a high viscosity and fast curing silicone (BodySil, Smooth-On) to seal all the interfaces present between the assembled pieces completely.

We used a silicone blend formulated in-house to mould the haptic liver parenchyma. This is constituted by a poorly cross-linked tacky dielectric gel (10 Shore000 hardness), mixed with a 30 ShoreA platinum catalysed hardener with brittle properties at different ratios. The relative volumetric amounts between the two components were empirically set by validating bulky moulded samples (total pre-polymer volume between 250 mL and 500 mL) with qualitative mechanical testing. More specifically, the specimens were systematically subjected to mechanical stimuli of relevance in surgery (e.g., laceration in cuts and incisions made with medical scalpels, elastic springback on palpation, compliance, and brittleness under pressure stimuli) to evaluate their functional response. We identified that the best formulation to reproduce the haptic response of the liver parenchyma, resembling that of an adult individual, was dielectric gel: hardener = 1:0.1 *v*/*v*. The curing time of the silicone blend is around 24 h at room temperature, after which demoulding took place to obtain the liver models.

#### 2.3.4. Evaluation Methods

The evaluation method was developed as a part of our ongoing clinical trial to evaluate the potential benefit of the 3D liver models in different clinical settings. The different prototypes prepared with the described approach were evaluated by an expert panel of 12 experts (6 HB surgeons and 6 radiologists) who assessed the accuracy of the model. A specific evaluation was requested by giving four possible scores: unsatisfactory (score 0), satisfactory (score 1), good (score 2), and excellent (score 3). The clinical evaluation score was requested to each expert for three different scenarios: (a) by improving the comprehension of the anatomical details when the model was compared with CT scan and MRI; (b) giving better information of the spatial relationship of venous, arterial, and biliary tree branching when compared with the standard imaging techniques (c) evaluation of the haptic properties by digitoclasic simulation of the 3D printed liver.

## 3. Results

**Figure 3:**
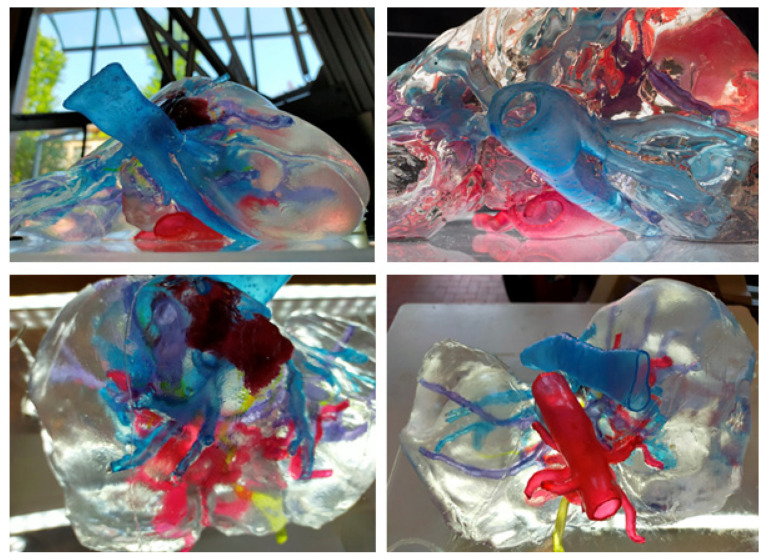
The optimised liver model with a more detailed intra-parenchymal arterial, portal and outflow venous branching of the biliary tree and intrahepatic lesion with a high-quality haptic perception.

A 3D transparent liver model was developed, refined, and optimised during different stages of evaluation of its haptic properties assessing the reliability of the liver anatomy as a possible exercise of preoperative surgical planning and simulation utilising the different surgical approaches utilised by the HB surgical team. We obtained an optimised haptic liver model with a more detailed intra-parenchymal arterial, portal, and outflow venous branching of the biliary tree and intrahepatic lesion with a high-quality haptic perception. Images of the definitive haptic models are reported in Figure 3.

Each part of the vascular anatomy (venous outflow, portal venous system, and arteri-al branching) was independently placed within the transparent liver parenchyma, which eventually led to the fully assembled model embedding the intrahepatic biliary tree and hepatic lesion. The reproduction of the parenchyma, using the transparent tissue-equivalent silicone developed in-house, enabled the direct visualisation of the integrated vasculo-biliary tree and an intra-parenchymal lesion. The different structures could be clearly identified thanks to their different colours preserving the spatial configuration of the anatomy and morphology.

The result of the evaluation from the expert panel of HB surgeons and radiologists is reported in Table 1, with an overall score ranging from 2.0 (good quality) to 3.0 (excellent quality). The haptic phantom was effective in providing a comprehensive overview of the anatomical details and the spatial relationships of the intrahepatic hematoma. The expert team of 12 radiologists and surgeons evaluated excellent the 3D anatomical details of the transparent definitive liver models as it allowed clear visualisation of the structures of the porta hepatic and intrahepatic anatomy from any point of view.

In particular, the model integrated the traditional visualisation of CT scans and MRI imaging by offering unlimited viewing angles of the liver anatomy and the possibility to show deformation of the liver structures when compression was applied during manipulation and rotational manoeuvres of the liver lobes, with the consequent changing pattern of the vascular-biliary tree and venous outflow.

## 4. Discussion

The liver is unique in that it receives a dual blood supply with double arterial and venous inflow and a unique venous outflow. Based on the concept of functional liver anatomy developed by Couinaud and Bismuth [35,36], identifying of hepatic structures is crucial to avoid liver damage during hepatic resection to spare as much as possible disease-free liver parenchyma. At present, this relies almost entirely on the accurate preoperative evaluation by CT scan and MRI, and intraoperative ultrasound, which requires a significant effort of abstract deduction of the 3D spatial relationships between intrahepatic structures.

During anatomical hepatic resection, the approach to the regional glissonian pedicle can be achieved by different techniques depending on the different approaches utilised, from open surgery to minimally invasive surgery such as video laparoscopy or robotic-assisted surgery. Whichever technique is used, the mobilisation and rotation of the liver during surgical manoeuvres modify the spatial relationship between the glissonian pedicle and the surface of the liver; these positional changes are difficult to imagine based on 2D CT scan images alone. Moreover, the intrahepatic glissonian and hepatic veins are intricately crossed, and the identification of each structure is difficult even with intraoperative navigation methods like ultrasound guidance because of the intrinsic noise of this imaging technique. Our haptic transparent phantom offers the possibility to visualise the intricate vasculo-biliary anatomy and to provide a unique understanding of the complex relationship between the liver vasculature and eventual intra-parenchymal lesions, like a subcapsular liver hematoma of the right lobe in our case. Moreover, the use of materials with haptic properties allows a tactile sampling of different portions of the liver and to appreciate the changing pattern of the intrahepatic structures during different manipulation of the liver, which can offer surgeons an effective tool for optimal preoperative evaluation and may help to select the best surgical strategy [37].

Other solutions for the visualisation of the intra-parenchymal anatomy of the liver using life-sized phantoms have been proposed in the literature. For example, the use of echogenic materials for the fabrication of the model parenchyma in conjunction with an ultrasound simulator has been recently demonstrated [38]. This approach enabled the possibility to simulate echography-guided procedures, reproducing the characteristic ultrasound imaging of the organ, but it could not provide the direct visualisation of the vasculo-biliary tree due to the opacity of the parenchyma. An alternative interesting strategy relies on the use of haptic augmented reality (AR) platforms [39]. In hepatic surgery, the adoption of AR provides the visualisation of the intra-parenchymal structures by super-imposing the 3D digital models on the real organ, phantoms or on laparoscopic images [40]. This, for example, offers the possibility to locate tumours that are difficult to be visualised by means of intraoperative imaging, such as laparoscopic ultrasonography. However, when AR navigation is used in hybrid simulators with phantoms, the haptic feedback of the physical model remains the crucial aspect that provides a reliable and accurate simulation experience of the surgical procedure (e.g., hepatic resections and biopsy). Furthermore, the employment of a transparent phantom, such as the one presented in this work, does not require the implementation of markers on the model for AR images alignment and system calibration, since the 3D visualisation of the intra-parenchymal anatomy is direct.

From the design and fabrication point of view, the examples of multi-coloured 3D liver prototypes with adequate parenchyma transparency for surgical planning evaluation relied either on the direct printing of the full model using PolyJet printing [31] or on the 3D assembly of the different structures combining standard 3D printing and silicone moulding [41]. In the first case, partially hollow vessels monolithically embedded in transparent parenchyma can be manufactured with high dimensional accuracy, but the mismatch between the printed parts and native tissues in terms of haptic feedback is relevant, in particular for what concerns the parenchymal structure [37]. Furthermore, multi-material printing with such technology presents high production and raw materials costs. In the hybrid approach, the manufacturing of the hepatic-biliary tree and intra-parenchymal pathologies, whether present, is based on standard materials such as poly lactic acid (PLA) or acrylonitrile butadiene styrene (ABS) by means of FFF or laser sintered thermoplastics.

These low costs polymers present ease of fabrication with desktop printers and acceptable dimensional resolution with respect to the anatomical details to reproduce but dramatically fail in replicating the haptic response of the native tissues due to the significant characteristic stiffness of thermoplastics. In this sense, common procedures in hepatic surgery, such as hepatic resections and anastomosis, cannot be performed on this type of phantoms since they require the use of soft tissue-mimicking materials to simulate the tasks adequately. Moreover, the vascular ducts are fabricated as solid parts representing only the lumen of the vessels [42]. Examples of liver models fabricated using 3D moulding have been demonstrated with both silicones and water-based physical gels to mimic the haptic response of the liver parenchyma, with embedded vascularization and biliary duct [43,44]. The use of soft polymeric materials to simulate the parenchymal physical properties targets at reproducing the average liver hardness, which, according to biomechanical data, ranges between 50 Shore000 and 80 Shore00 [44]. The silicone-based materials employed for this purpose exhibit larger hardness (higher than 10 ShoreA) and are often opaque or translucent polymers that do not allow the correct visualisation of intra-parenchymal structures [1,45]. In contrast, physical gels based on polyvinyl alcohol, phytagel and agarose possess adequate hardness and transparency but offer a limited lifetime due to the presence of water incorporated into the polymers or must undergo time-consuming and labour-intensive freeze-thawing cycles to obtain the final organ model with a suitable haptic response [44].

The liver model proposed in this work simulates the overall haptic feedback of the native organ with high fidelity due to the combined use of hollow vascular structures based on soft photopolymers and the developed transparent tissue-mimetic silicone blend representing the parenchyma, engineered with optimised density and functional response under mechanical stimuli relevant in HB surgery.

## 5. Conclusions

We developed a hybrid approach to fabricate a patient-specific 3D life-sized transparent liver model with haptic properties from standard CT scans and MRI, including eventual intra-parenchymal lesions. The images were processed using a semiautomatic procedure for segmentation and co-registration to generate the 3D digital models of the target anatomical structures. These were used as the source data to produce the physical liver model, fabricated by combining 3D printing and moulding of tissue-mimicking materials.

Our 3D transparent model, fabricated with soft materials resembling the properties of living tissues, has been evaluated as able to improve surgeons’ understanding of the positional changes of intrahepatic structures during the surgical procedure and the relationship with the liver lesion during manipulation. Therefore, it can be considered a possible implementation of the surgical diagnostic ability of the virtual reality concept for correct preoperative surgical planning, an effective simulation tool for medical training and education, with a real possibility to improve the safety of complex liver surgery.

## Figures and Tables

**Table 1:** Evaluation score by the expert team composed of six HB surgeons and six radiologists.

	Anatomical Details	Spatial Relation of the Venous and Vasculo-Biliary Three	Haptic Properties
HB surgical team score (total/mean)	18/3	16/2.7	12/2.0
Expert radiology team score (total/mean)	18/3	15/2.5	13/2.2

## Data Availability

Not applicable.
